# ER, PgR, Ki67, p27^Kip1^, and histological grade as predictors of pathological complete response in patients with HER2-positive breast cancer receiving neoadjuvant chemotherapy using taxanes followed by fluorouracil, epirubicin, and cyclophosphamide concomitant with trastuzumab

**DOI:** 10.1186/s12885-015-1641-y

**Published:** 2015-09-07

**Authors:** Sasagu Kurozumi, Kenichi Inoue, Hiroyuki Takei, Hiroshi Matsumoto, Masafumi Kurosumi, Jun Horiguchi, Izumi Takeyoshi, Tetsunari Oyama

**Affiliations:** 1 Division of Breast Surgery, Saitama Cancer Center, Saitama, Japan; 2 Division of Breast Oncology, Saitama Cancer Center, Saitama, Japan; 3 Department of Pathology, Saitama Cancer Center, 780 Komuro, Ina-machi, Kitaadachi-gun, Saitama 362-0806 Japan; 4 Department of Thoracic and Visceral Organ Surgery, Gunma University Graduate School of Medicine, Maebashi, Gunma Japan; 5 Department of Diagnostic Pathology, Gunma University Graduate School of Medicine, Maebashi, Gunma Japan

## Abstract

**Background:**

Neoadjuvant chemotherapy (NAC) with taxanes followed by fluorouracil, epirubicin, and cyclophosphamide (FEC), and concurrent trastuzumab is a potent regimen for HER2 over-expressing breast cancer. A high pathological complete response (pCR) rate has been achieved using this regimen; however, the predictive factors and prognostic effects of pCR currently remain unclear. In the present study, we determined whether pCR was related to histological grade (HG) and several biological factors including p27^Kip1^. We also assessed the prognosis of the pCR and non-pCR groups, and expected differences between those positive and negative for lymph node metastasis after chemotherapy.

**Methods:**

A total of 129 Japanese women with HER2-positive invasive breast cancer received either paclitaxel or docetaxel followed by FEC, with the concomitant administration of trastuzumab. The statuses of HG, ER, PgR, Ki67, and p27^Kip1^ were evaluated to determine their relationship with pCR. Relapse-free survival (RFS) and overall survival (OS) were also analyzed for their relationship with pCR and pathological nodal involvement.

**Results:**

pCR was obtained in 84 out of 129 patients and the pCR rate was 65.1 %. The pCR rates related to 5 factors were as follows: HG (grade 3, 70.0 % vs. grades 1–2, 36.8 %), ER (negative, 78.6 % vs. positive, 40.0 %), PgR (negative, 75.3 % vs. positive, 38.9 %), Ki67 (high, 72.0 % vs. low, 47.2 %), and p27^Kip1^ (low, 71.0 % vs. high, 50.0 %). RFS was significantly better in the pCR group than in the non-pCR group (*p* = 0.018). Patients with remaining nodal disease in the pCR group had worse OS (*p* = 0.0002).

**Conclusions:**

High-HG, low-ER, low-PgR, high-Ki67, and low-p27^Kip1^ were identified as predictive factors of pCR in NAC with trastuzumab, while pCR and negative nodes were predictive of better survivals.

**Electronic supplementary material:**

The online version of this article (doi:10.1186/s12885-015-1641-y) contains supplementary material, which is available to authorized users.

## Background

Many neoadjuvant chemotherapy (NAC) regimens for breast cancer that use various cytotoxic agents are currently being performed in clinical studies as well as routine practice, and NAC is considered a useful therapeutic option for advanced as well as early stage breast cancer patients. NAC improves surgical outcomes in advanced cases for which mastectomy is technically impossible and in early cases of operable breast cancer desiring breast conservative surgery. The therapeutic effects of NAC have mainly been evaluated on the basis of the pathological findings and results of most clinical studies including NSABP protocol B-18 [1] and B-27 [2]. These studies confirmed the prognostic significance of pathological complete response (pCR). On the basis of these findings, pCR has been positioned as a primary endpoint of NAC in many clinical studies and clinical trials of new chemotherapeutic agents.

Approximately 20 to 30 % of breast cancers over express human epidermal growth factor receptor 2 (HER2) [3], and effective HER2-targeting agents such as trastuzumab have recently been added to NAC regimens [4–6]. However, the pCR rate of NAC using taxanes and trastuzumab has remained between 20 and 40 % [4, 7]. An anthracycline has also been added to NAC regimens with trastuzumab in an attempt to obtain a higher pCR rate. Buzdar et al. [8] conducted a NAC regimen with tri-weekly paclitaxel followed by fluorouracil, epirubicin, and cyclophosphamide (FEC) concurrently with trastuzumab on HER2-positive patients and achieved a pCR rate of 66.7 % without serious adverse events. However, the predictive factors of pCR in patients with HER2-positive breast cancer receiving trastuzumab supplemented NAC regimens have not been clarified.

In the present study, we analyzed several factors, such as the histological grade (HG), ER status, PgR status, Ki67 (proliferation marker) labeling index (LI), and expression of p27^Kip1^ (an anti-proliferation marker) in order to identify more effective predictive markers for pCR after NAC with trastuzumab. We also assessed differences in survival between the pCR and non-pCR groups, and between patients who tested positive and negative for lymph node metastasis.

## Methods

### Patient backgrounds and eligibility

Our study included 129 female patients with HER2-positive invasive breast cancer. They received NAC consisting of 12 cycles of paclitaxel (80 mg/m^2^) every week or 4 cycles of docetaxel (75 mg/m^2^) every 3 weeks followed by 4 cycles of FEC-75 (5-fluorouracil, 500 mg/m^2^, epirubicin, 75 mg/m^2^; and cyclophosphamide, 500 mg/m^2^) every 3 weeks. All patients also received 4 mg/kg trastuzumab on day 1 of the treatment and 2 mg/kg trastuzumab every week thereafter for a total of 24 cycles. NAC with trastuzumab for these patients was performed to evaluate the impact of pathological therapeutic effects evoked by additional administration of trastuzumab, and the main aims of this therapy for each patient were reduction of tumor size for prior to breast-conserving surgery, and evaluation of drug effect through pathological assessment of tumor response and good prognosis in cases achieving pCR.

The main eligibility criteria were: age ≥20 years, an Eastern Cooperative Oncology Group performance status of 0–1, adequate oral intake, preserved major organ functions, and the ability to provide informed consent. Patients were excluded if they had a previous history of therapy for breast cancer, inflammatory breast cancer, a history of severe anaphylaxis or allergies to any drug, significant active illness that may preclude the protocol treatment, a history of uncompensated congestive heart failure, or severe mental disease. Pregnant or lactating females were also excluded. This protocol is now accepted as standard care. Approximately 97 % of patients completed this regimen of treatment. However, some patients receiving the same regimen of treatment were excluded from this study because their paraffin-embedded pre-treatment biopsy specimens could not be obtained. In approximately 40 % of patients, sentinel lymph node biopsy was performed using a combination method with an isotope (99mTc-sulfur colloid) and blue dye.

We extracted information from the institutional clinical and pathological database on all consecutive breast cancer patients who underwent surgery between 2005 and 2011 at the Division of Breast Surgery in the Saitama Cancer Center. All patients received an adjuvant trastuzumab treatment for six months, and adjuvant endocrine therapy using tamoxifen or an aromatase inhibitor was performed on ER-positive patients. This study was conducted in accordance with the Declaration of Helsinki, and the protocol of NAC used in this study and associated translational researches were approved by the Institutional Review Board of the Saitama Cancer Center. All patients enrolled in this study agreed to be treated with this NAC regimen and provided written informed consent.

### Evaluation of histological effects

Pathological effects after NAC were evaluated by 2 pathologists according to the criteria proposed by the Japanese Breast Cancer Society (JBCS). In these criteria, pathological effects were categorized into 6°, such as grade 0, 1a, 1b, 2a, 2b, and 3, on the basis of morphological changes and the extent or absence of invasive cancer. Grade 1a was defined as “mild changes in cancer cells regardless of the extent, and/or marked changes in <1/3 of the tumor”. Grade 1b was defined as “marked changes in 1/3 to <2/3 of the tumor”. Grade 2a was defined as “marked changes in ≥2/3 of the tumor, but apparent remaining cancer cells”. Grade 2b was defined as “marked changes approaching a complete response with only a few remaining cancer cells”. The JBCS defines “grade 3” as “no invasive cancer in the breast”, which is equal to “pCR” proposed in the NSABP B-18 study. The presence of non-invasive cancers (DCIS) in the breast and nodal involvement in such cases was clearly stated. This response was designated as ypT0/is ypN0/1-3 according to the TNM staging criteria of the AJCC (American Joint Committee on Cancer Staging System).

### Examination of biological markers in relation to pCR rates

**Fig. 1:**
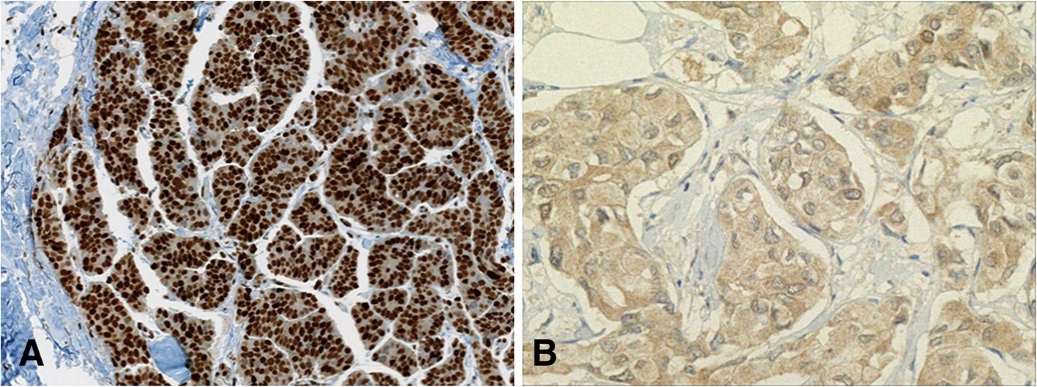
Immunohistochemical findings of p27^Kip1^_._ **a** Nuclei of cancer cells showing highly positive immunoreactions for p27^Kip1^. **b** The cytoplasm of cancer cells was weakly positive, whereas the nuclei were negative for p27^Kip1^

The expression of ER, PgR, and HER2 proteins was examined by immunohistochemistry and HER2 gene amplification was evaluated by fluorescence *in situ* hybridization (FISH) using specimens obtained by needle biopsy as a routine practice before NAC. On the other hand, the expression of Ki67 and p27^Kip1^ was retrospectively examined by immunohistochemistry using needle biopsy specimens as part of this study. Primary antibody sources were as follows: ER (1D5, DAKO, Denmark), PgR (PgR636, DAKO, Denmark), HER2 (HercepTest, DAKO, Denmark), Ki67 (MIB-1, DAKO, Denmark), and p27^Kip1^ (Santa Cruz Biotechnology, USA). Positive ER and PgR statuses were defined by the presence of 1 % or more positive nuclei. A high Ki-67 LI and strong expression of p27^Kip1^ were defined by the presence of 30 % and 75 % or more positive nuclei, respectively (Fig. 1a). Cancer cells that positively expressed p27^Kip1^ in the cytoplasm were categorized as “negative” (Fig. 1b). The ER status (positive vs. negative), PgR status (positive vs. negative), Ki67 LI (high vs. low), and p27^Kip1^ expression (high vs. low) at baseline were analyzed for their relationships with pCR.

### Clinical outcome analysis

Clinical and tumor characteristics at baseline such as age, menopausal status, clinical tumor size, clinical nodal status, and HG (grades 1–2 vs. grade 3) were analyzed for their relationship with pCR and with the presence of pathological axillary lymph node metastasis. Recurrence-free survival (RFS) and overall survival (OS) were compared according to the achievement of pCR and pathological nodal involvement.

### Statistical analysis

Statistical analyses were conducted using Stat Mate 4 for Windows (ATMS, Tokyo, Japan). The Chi-squared test and Fisher’s exact test were used to analyze relationships between clinicopathological characteristics and pCR. In addition, a multivariate analysis of logistic regression was used to determine which factors were independently associated with pCR.

The Kaplan-Meier method and log-rank test were used to estimate RFS and OS rates. RFS was defined as the length of time from the period of surgery to any recurrence (including ipsilateral breast recurrence). OS was determined as the time from the day of surgery until the time of death (from any cause). The log-rank test was used to compare survival rates between patients with pCR and those with non-pCR. Survival rates were analyzed for their relationship with pathological nodal involvement in the pCR group.

## Results

### Patient and tumor characteristics

The median age of the 129 patients enrolled in this study was 53 years (age range, 27–73 years); 109 patients (84.5 %) were older than 41 years and 78 patients (60.5 %) were post-menopausal. Tumor sizes (AJCC) were as follows: T1, 6 patients (4.7 %); T2, 81 patients (62.8 %); T3, 26 patients (20.2 %); and T4, 16 patients (12.4 %). The clinical lymph node status was as follows: N0, 41 patients (31.8 %); N1, 59 patients (45.7 %); N2, 17 patients (13.2 %); and N3, 12 patients (9.3 %). Breast cancer stages were as follows: stage I, 2 patients (1.6 %); stage IIA, 37 patients (28.7 %); stage IIB, 38 patients (29.5 %); stage IIIA, 29 patients (22.5 %); stage IIIB, 11 patients (8.5 %); and stage IIIC, 12 patients (9.3 %). In 38.8 % of all cases, lymph node dissection was avoided, because of negative for metastasis in sentinel lymph nodes (Additional file 1); the median number of nodes removed by axillary lymph node dissection was 11 (range, 2–23) , and he median number of nodes sampled only by sentinel lymph node biopsy was 2 (range, 1–5).

### Tumor response

Eighty-four (65.1 %) out of the 129 patients achieved pCR (grade 3, JBCS criteria). Of these, 5 patients (3.9 %) had lymph node metastasis (ypN1-3) and 31 (24.0 %) had DCIS. The histological tumor responses of the 45 patients who did not achieve pCR (non-pCR) were as follows: grade 2b (near-pCR), 11 patients (8.5 %); grade 2a, 17 patients (13.2 %); grade 1b, 12 patients (9.3 %); and grade 1a, 5 patients (3.9 %). A prominent tumor response (pCR and near-pCR) was detected in 73.6 % of patients.

### Patient and tumor characteristics and biological markers associated with pCR

pCR rates were not associated with clinical T (*p* = 0.62), clinical N (*p* = 0.96), or clinical stages (*p* = 0.97); however, pCR rates correlated with the menopausal status (*p* = 0.019) (Table 1). pCR rates were also significantly higher in patients with HG 3 (*p* = 0.005), ER-negative (*p* < 0.001), and PgR-negative (*p* < 0.001) tumors than in those with HG 1–2, ER-positive, and PgR-positive tumors, respectively (Table 1). pCR rates were also significantly higher in patients whose tumors had a high Ki67 LI (*p* = 0.008) or weakly expressed p27^Kip1^ (*p* = 0.025) (Table 1). The multivariate analysis indicated that none of the factors were significant (Additional file 2). Therefore, the relationship between pCR rates and the 5 biological markers (HG, ER, PgR, Ki67, and p27^Kip1^) combined was evaluated. One point each was assigned for high HG, ER negative, PgR negative, high Ki-67 LI, and low p27^Kip1^ expression. pCR rates among patients with 5, 4, 3, 2, 1, and 0 points were 84.3 %, 74.1 %, 47.8 %, 47.1 %, 28.6 %, and none, respectively. This pCR-predicting score significantly predicted pCR (*p* = 0.0001) (Table 2 and Additional file 3).

**Table 1:** Patient and tumor characteristics at baseline and their relationship with pCR

	pCR	non-pCR	pCR rate (%)	*p*-value
Menopausal status				
Premenopausal	27	24	52.9	
Postmenopausal	57	21	73.1	0.019*
Clinical tumor size				
T1	4	2	66.7	
T2	56	25	9.1	
T3	15	11	57.7	
T4	9	7	56.2	0.62
Clinical nodal status				
N0	27	14	65.9	
N1	39	20	66.1	
N2	11	6	64.7	
N3	7	5	58.3	0.96
Clinical stage				
I	1	1	50.0	
IIA	26	11	70.3	
IIB	24	14	63.2	
IIIA	19	10	65.5	
IIIB	7	4	63.6	
IIIC	7	5	58.3	0.97
Type of Taxanes				
Paclitaxel	78	39	66.7	
Docetaxe	6	6	50.0	0.40
HG				
High (3)	77	33	70.0	
Low (1–2)	7	12	41.2	0.005*
ER				
Negative (<1 %)	66	18	78.6	
Positive (≥1 %)	18	27	40.0	<0.001*
PgR				
Negative (<1 %)	70	23	75.3	
Positive (≥1 %)	14	22	38.9	<0.001*
Ki67				
High (≥30 %)	67	26	72.0	
Low (<30 %)	17	19	47.2	0.008*
p27^Kip1^				
Low (<75 %)	66	27	71.0	
High (≥75 %)	18	18	50.0	0.025*

*pCR*, pathological complete response; *HG*, histological grade; *ER*, estrogen receptor; *PgR*, progesterone receptor

*p < 0.05 was considered significant; all significant values are shown in bold

**Table 2:** pCR prediction scores and their relationship with pCR

Scores	pCR	Non-pCR	pCR rate (%)	p-value
0	0	4	-	
1	2	5	28.6	
2	8	9	47.1	
3	11	12	47.8	
4	20	7	74.1	
5	43	8	84.3	0.0001*

One point each was assigned for high HG, ER negative, PgR negative, high Ki-67 LI, and low p27^Kip1^ expression

pCR, pathological complete response

*p < 0.05 was considered significant; significant values are shown in bold

### Survival

**Fig. 2:**
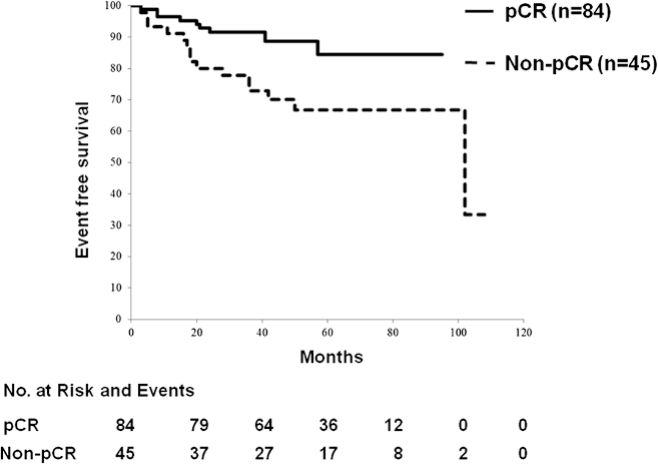
Relapse-free survival curve of pCR and non-pCR patient groups. RFS was significantly better in patients with pCR after neoadjuvant therapy than in those without pCR (HR = 0.40, *p* = 0.012)

**Fig. 3:**
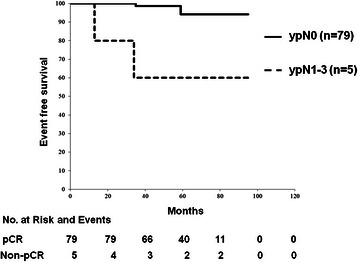
Overall survival curve of pCR patients with and without lymph node metastasis. In pCR cases, OS was significantly better in patients without lymph node metastasis than in those with lymph node metastasis (HR = 13.34, *p* = 0.0002)

The median RFS was 53 months (range, 3–108 months) and median follow-up duration was 59 months (range, 13–108 months). RFS was significantly better in the 84 patients with pCR after NAC with trastuzumab than in the 45 patients with non-pCR [hazard ratio (HR) = 0.40, 95 % CI = 0.15 to 0.79, *p* = 0.012] (Fig. 2). Of the 84 patients with pCR, OS was significantly worse in those with pathologically involved nodes (ypT0/is ypN1-3) (5 patients) than in those without pathologically involved nodes (ypT0/is ypN0) (79 patients) (HR = 0.075, 95 % CI = 0.019 to 0.29, *p* = 0.0002) (Fig. 3). Among patients with ER-positive tumors, pCR was significantly predictive of better RFS if the tumors had a high Ki67 LI (*p* = 0.004); however, pCR was not predictive of RFS rates if tumors showed a low Ki67 LI. pCR was significantly predictive of better RFS rates in patients with tumors revealing a high Ki67 LI (HR = 0.036, 95 % CI = 0.085 to 0.82, *p* = 0.021), but not in patients with tumors showing a low Ki67. pCR was predictive of better RFS in patients with tumors that weakly expressed p27^Kip1^, (HR = 0.35, 95 % CI = 0.094 to 0.72, *p* = 0.010), but not in patients with tumors that strongly expressed p27^Kip1^.

## Discussion

In the present study, we clarified that NAC with taxanes followed by FEC concurrently with trastuzumab was useful for obtaining a high pCR rate of more than 65 % and the statuses of high HG, low ER, low PgR, high Ki67, and low p27^Kip1^ appeared to be predictive factors for a high pCR rate. Furthermore, prognoses were significantly better in patients with pCR and pathologically negative lymph nodes after NAC with trastuzumab.

Trastuzumab, a humanized monoclonal antibody to the HER2 protein, binds to the extracellular domain of HER2, which is localized in the cell membrane of carcinoma cells, and inhibits their proliferation by arresting the cell cycle during the G1 phase [9, 10]. Trastuzumab has been suggested to inhibit HER2 action by preventing HER2 dimerization and subsequent signal transduction via the phosphatidylinositol 3-kinase (PI3K) cascade [11, 12]. It also causes antibody-dependent, cell-mediated cytotoxicity, and its binding to cancer cells triggers their killing by immune cells [13, 14]. Since trastuzumab was accepted as a new agent for breast cancer patients with HER2 over-expressing tumors by the Food and Drug Administration (FDA) in 1998, the strategy of breast cancer treatment has markedly changed in the last decade.

Preclinical models suggested that the concurrent administration of trastuzumab and chemotherapy was synergistic [15–17], and Siedman et al. [18] reported that chemotherapy using trastuzumab (2 mg/kg) and paclitaxel 90 mg/m^2^ was clinically effective for metastatic breast cancer patients with HER2-positive tumors, with overall response rates ranging between 67 and 81 %. Trastuzumab has also been used in adjuvant therapy, and Smith et al. [19] reported that a 1-year treatment with trastuzumab after adjuvant chemotherapy in the HERA study led to significant overall survival benefits. When administered concurrently with NAC, trastuzumab was shown to markedly improve pCR rates in patients with HER2-positive tumors [4, 7]. Buzdar et al. [8, 20] also reported a pCR rate of 66.7 % in NAC with tri-weekly paclitaxel followed by FEC concurrently with trastuzumab. Our modified regimen, such as “weekly paclitaxel instead of tri-weekly or tri-weekly docetaxel”, also achieved a pCR rate of 65.1 %. However, the pCR rate decreased to 61.2 % when the 5 patients with pathologically involved nodes were excluded. In addition, the foci of DCIS remained in 24.0 % of cases. Therefore, DCIS may be less responsive to this kind of therapy because the carcinoma cells of DCIS are localized in pre-existing lactiferous ducts without feeding vessels.

Few studies have attempted to identify the predictive markers of pCR after NAC in patients with HER2-positive tumors [21, 22]. In our translational research to obtain higher pCR rates, we clarified the relationship between pCR rates and the statuses of several biological factors. The negative expression of hormone receptors (ER and PgR) may be important for predicting pCR in NAC with trastuzumab. The pCR rate of the ER-negative group (78.6 %) was higher than that of the ER-positive group (40.0 %), and was also higher in the PgR-negative group (75.3 %) than in the PgR-positive group (38.9 %). Furthermore, an evaluation of the high proliferation marker (Ki67) LI was considered to be useful for estimating pCR, with the pCR of the high Ki67 (≥30 %) group (72.0 %) being higher than that of the low Ki67 group (47.2 %). An estimation of HG may also be useful for the selection of high pCR patients because the pCR rate of the high HG (grade 3) group was 70.0 % while that of the low HG group (grade 1–2) was 36.8 %.

On the other hand, p27^Kip1^ is a cyclin dependent kinase inhibitor that normally acts as a tumor suppressor protein [23–26]; however, the relationship between the expression of p27^Kip1^ and pCR in NAC with trastuzumab has not yet been ascertained. Previous studies suggested that breast cancers over-expressing HER2 become resistant to trastuzumab by activating the PI3K/Akt signaling pathway and downregulating p27^Kip1^ [27, 28]. Activated Akt phosphorylates p27^Kip1^, which may result in the mislocation of p27^Kip1^ to the cytoplasm, in which it is unable to inhibit cell cycle proteins; nuclear localization is important for its cell cycle inhibitory function [29]. Low amounts of p27^Kip1^ in the nucleus and resultant activation of cyclin-dependent kinases in the nucleus may increase the proliferation of tumor cells [30, 31]. Difficulties are associated with evaluating the expression of p27^Kip1^ because 3 potential expression patterns exist: only in the nucleus, only in the cytoplasm, and in both the nucleus and cytoplasm [32, 33]. Stendahl et al. [34] previously defined grade 3 of p27^Kip1^ as 75 % or more cancer cells with p27^Kip1^-positive nuclei. By using the same cut-off (75 %) in the present study, we found that the weak expression of p27^Kip1^ was predictive of pCR (low, 71.0 % vs. high, 50.0 %).

The results of the present study indicated that a set of 5 factors (HG, ER, PgR, Ki67, and p27^Kip1^) more accurately predicted pCR than each factor individually. pCR prediction scores of 0 to 5, which were calculated based on the presence of a combination of these factors, predicted pCR rates of 0 % to 84.3 %. Prospective studies are needed to determine whether pCR prediction scores accurately predict pCR.

As reported in recent clinical trials on HER2-positive breast tumors [6, 35], pCR after NAC with HER2-targeted agents may be a surrogate marker of prognosis; however, our results indicated that RFS was significantly better in patients with pCR after NAC with trastuzumab than in those with non-pCR. This result was consistent with previous findings reported by the NSABP B-18 [2] and B-27 [3] studies. In the NSABP B-27 trial, patients without nodal involvement had significantly better OS rates than those with nodal involvement if nodal involvement was the only parameter analyzed, and OS rates decreased as the number of involved nodes increased [2]. As noted above, recent studies suggest that pCR criteria include the lack of nodal involvement (e.g., “ypT0 ypN0” and “ypTis ypN0”) [36]. In the present study, nodal involvement was significantly predictive of low OS rates in patients with pCR. Three out of the 5 patients with pCR and nodal involvement had recurrent tumors; 2 were HER2-negative residual tumors in the axillary nodes. Differences in the expression of HER2 between primary breast tumors and metastatic tumors in regional nodes or at distant sites may reflect the heterogeneous characteristics of tumors.

HER2-positive tumors are ER-positive (designated luminal B-like, HER2-overexpressing) or ER-negative (designated non-luminal, HER2-enriched) [37]. In our study, pCR rates were significantly higher in patients with non-luminal-like tumors than in those with luminal-like tumors. Cross-talk between the ER and HER2 signaling pathways in luminal-like tumors may be responsible for the lower response of these tumors to NAC with trastuzumab [38]. pCR was previously reported to be predictive of high survival rates in most patients with breast cancer, but not in patients with luminal-like tumors [22, 39]. However, as demonstrated in the present study, pCR was predictive of RFS rates, even in patients with luminal-like breast cancer if their tumors had a high Ki67 LI (≥30 %). Therefore, pCR in combination with a high Ki67 LI at baseline may be predictive of survival rates in patients with luminal-like, HER2-overexpressing breast cancer.

## Conclusions

In the present study, we confirmed that NAC using taxanes followed by FEC concurrent with trastuzumab was a useful regimen for obtaining a high pCR rate of more than 65 %. By histopathological and immunohistochemical evaluations, we identified high HG, ER-negative, PgR-negative, high Ki67 LI, and low p27^Kip1^ as significant predictive factors of pCR. In addition, significantly better prognoses were achieved in the pCR group and pathologically negative lymph node group after this regimen of NAC with trastuzumab.

## Additional files

Additional file 1: **Patient and tumor characteristics at baseline.** (PDF 6 kb)
Additional file 2: **Results of the multivariate analysis of clinicopathological factors influencing the prediction of pCR.** (PDF 47 kb)
Additional file 3: **pCR prediction scores and their relationship with pCR (odds ratio and 95 % confidence intervals).** (PDF 17 kb)

## Abbreviations

NAC: neoadjuvant chemotherapy
HER2: human epidermal growth factor receptor 2
FEC: fluorouracil, epirubicin and cyclophosphamide
pCR: pathological complete response
RFS: relapse-free survival
OS: overall survival
LI: labeling index
JBCS: Japanese Breast Cancer Society
DCIS: noninvasive cancers
AJCC: American Joint Committee on Cancer Staging System
FISH: fluorescence *in situ* hybridization
PI3K: phosphatidylinositol 3-kinase
FDA: Food and Drug Administration
HR: hazard ratio
