# Supplementary material for: ER, PgR, Ki67, p27Kip1, and histological grade as predictors of pathological complete response in patients with HER2-positive breast cancer receiving neoadjuvant chemotherapy using taxanes followed by fluorouracil, epirubicin, and cyclophosphamide concomitant with trastuzumab
Source: BMC Cancer. 2015 Sep 7;15:622. doi: 10.1186/s12885-015-1641-y (PMC4562359; doi:10.1186/s12885-015-1641-y)
Supplement: Additional file 1: — Patient and tumor characteristics at baseline. (PDF 6 kb) [file 12885_2015_1641_MOESM1_ESM.pdf]

Additional file 1. Patient and tumor characteristics at baseline

	No. of patients	%
Total	129	100
Menopausal status at diagnosis		
Premenopausal	51	39.5
Postmenopausal	78	60.5
Clinical tumor size		
T1	6	4.7
T2	81	62.8
T3	26	20.2
T4	16	12.4
Clinical nodal status		
N0	41	31.8
N1	59	45.7
N2	17	13.2
N3	12	9.3
Clinical stage		
I	2	1.6
IIA	37	28.7
IIB	38	29.5
IIIA	29	22.5
IIIB	11	8.5
IIIC	12	9.3
Type of surgery		
Breast-conserving surgery	109	84.5
Mastectomy	20	15.5
Axillary management		
Sentinel lymph node biopsy alone	50	38.8
Axillary lymph node dissection	79	61.2
Types of Taxanes		
Paclitaxel	117	90.7
Docetaxel	12	9.3
