# Supplementary material for: ER, PgR, Ki67, p27Kip1, and histological grade as predictors of pathological complete response in patients with HER2-positive breast cancer receiving neoadjuvant chemotherapy using taxanes followed by fluorouracil, epirubicin, and cyclophosphamide concomitant with trastuzumab
Source: BMC Cancer. 2015 Sep 7;15:622. doi: 10.1186/s12885-015-1641-y (PMC4562359; doi:10.1186/s12885-015-1641-y)
Supplement: Additional file 2: — Results of the multivariate analysis of clinicopathological factors influencing the prediction of pCR. (PDF 47 kb) [file 12885_2015_1641_MOESM2_ESM.pdf]

Additional file 2. Results of the multivariate analysis of clinicopathological factors influencing the prediction of pCR

Characteristics	HR	p-value	95% CI
HG			
Low (1-2)	Referent		
High (3)	2.00	0.25	0.62-6.42
ER			
Positive (≥1%)	Referent		
Negative (<1%)	3.04	0.061	0.95-9.76
PgR			
Positive (≥1%)	Referent		
Negative (<1%)	1.73	0.38	0.51-5.90
Ki67			
Low (<30%)	Referent		
High (≥30%)	2.37	0.063	0.95-5.89
$p27^{Kip1}$			
High (3)	Referent		
Low (1-2)	1.33	0.56	0.52-3.40

HR, hazard ratio; CI, confidence interval; pCR, pathological complete response; HG, histological grade; ER, estrogen receptor; PgR, progesterone receptor
*p<0.05 was considered significant; all significant values are shown in bold.
