# Supplementary material for: ER, PgR, Ki67, p27Kip1, and histological grade as predictors of pathological complete response in patients with HER2-positive breast cancer receiving neoadjuvant chemotherapy using taxanes followed by fluorouracil, epirubicin, and cyclophosphamide concomitant with trastuzumab
Source: BMC Cancer. 2015 Sep 7;15:622. doi: 10.1186/s12885-015-1641-y (PMC4562359; doi:10.1186/s12885-015-1641-y)
Supplement: Additional file 3: — pCR prediction scores and their relationship with pCR (odds ratio and 95 % confidence intervals). (PDF 17 kb) [file 12885_2015_1641_MOESM3_ESM.pdf]

Additional file 3. pCR prediction scores and their relationship with pCR (odds ratio and 95% confidence intervals)

Scores	pCR rate (%)	OR	95%CI
0	-	1.00	-
1	28.6	1.40	0.88-2.24
2	47.1	1.89	1.21-2.96
3	47.8	1.92	1.30-2.84
4	74.1	3.86	2.04-7.30
5	84.3	6.38	3.38-12.05

One point each was assigned for high HG, ER negative, PgR negative, high Ki-67 LI, and low $p27^{Kip1}$ expression.

pCR, pathological complete response; OR, odds ratio; CI, confidence interval
